# Gender-based violence during the COVID-19 pandemic response in Italy

**DOI:** 10.7189/jogh.10.020359

**Published:** 2020-12

**Authors:** Rebecca Lundin, Benedetta Armocida, Paola Sdao, Sigrid Pisanu, Ilaria Mariani, Antonella Veltri, Marzia Lazzerini

**Affiliations:** 1 Institute for Maternal and Child Health, IRCCS “Burlo Garofolo”, Trieste, Italy; 2 D.i.Re. Donne in Rete contro la violenza The Italian National Women’s Network against Violence, Rome, Italy

Gender-based violence (GBV), with one out of three women worldwide experiencing violence in their lifetime, has been defined by the World Health Organization (WHO) as a “global public health problem of epidemic proportions” [1]. During the current COVID-19 pandemic, the WHO and other international authorities have warned about the increased risk of GBV related to more time spent indoors, isolation from social and protective networks, and greater social and economic stress related to both the epidemic and response measures [2-4].

In fact, since the onset of the COVID-19 outbreak, reports from many countries including France, Germany, Spain, the United Kingdom, Cyprus, Argentina, Singapore, Canada, and the United States indicate that violence against women has increased [3-6].

In Italy, the most recent national data indicate that 31.5% of women between 16 and 70 years of age have experienced physical or sexual abuse at any point in their lives, with violence attributed to a current or former partner in 13.6% of cases [7]. After identification of the first COVID-19 case in Italy on 21 February 2020, social distancing measures were progressively enacted, culminating in a nation-wide lockdown which lasted about two months, from 11 March 2020 to 3 May 2020 [8]. As signatory of the Council of Europe Convention on preventing and combatting violence against women and domestic violence – known as the Istanbul Convention – Italy recognises GBV as a violation of human rights [9]. It is the obligation of the national government to fully address violence against women in all its forms and to take measures to prevent it, protect its victims and prosecute perpetrators. The state should also collect relevant disaggregated statistical data at regular intervals on cases of all forms of violence against women, and support research in the field in order to study its root causes and effects, incidence and conviction rates, as well as the efficacy of measures taken to implement this Convention [9].

**Figure 1:**
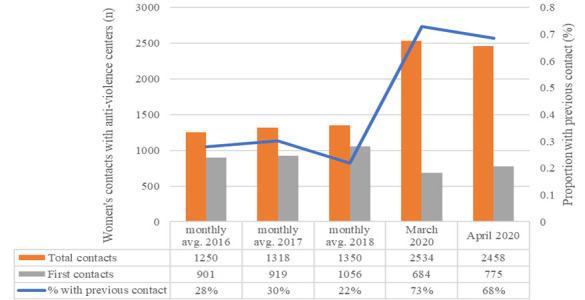
Women’s contacts with anti-violence centers in Italy, 2016-2020. Note: All 58 centres contributed data in each year shown. Monthly averages were extrapolated from annual data in 2016-2018, crude numbers reported from March and April 2020. Data not yet available for 2019.

We report here data from the Italian National Women’s Network Against Violence, D.i.Re. The network collects data annually from anti-violence centers which fulfill the Istanbul Convention minimum criteria for specialized service providers. During the COVID-19 pandemic network members were asked to review the number of women contacting anti-violence centers during two time periods, corresponding to about one month each (between 2 March and 5 April 2020 and between 6 April and 3 May 2020). Data from 58 centres during March 2020 and April 2020 were compared to monthly averages during the previous years (**Figure 1**). Participating centres were located in 16 out of Italy’s 20 regions, with more centres in Tuscany (n = 12), Emilia Romagna (n = 10), and Lombardy (n = 10), regions with, respectively, the 5th, 3rd, and 1st highest numbers of COVID-19 cases during the pandemic.

In this sample, while the monthly average of women accessing anti-violence services was relatively stable during the years 2016, 2017, and 2018 (mean value: 1306 women, 95% CI 1255 to 1357), a sharp increase was observed during March and April 2020 (mean value: 2496 women, 95%CI 2442 to 2550, +191,1% *P* < 0.00001). Interestingly, women with a history of previous contact with these anti-violence centres accounted for less than one third of cases during 2016, 2017, and 2018 (mean value: 27%, 95%CI 23% to 31%), but for over two-thirds of cases from March to April 2020 (mean value: 68%, 95%CI 64% to 72%, *P* < 0.00001).

These findings are in keeping with data published by the Italian National Institute of Statistics (ISTAT), indicating a 59% increase in calls to a hotline for GBV victims between 1 March and 16 April 2020 compared to the same period in 2019, with the vast majority of calls (93.2%) made by women with a long-term history of GBV [10]. Both the D.i.Re. network and the national government rolled out advertising campaigns on social and traditional media early in the COVID-19 pandemic, which may have increased awareness of their services. However, notably, both channels for the support of women at risk of or suffering GBV have consistently advertised their services in the past.

**Figure:**
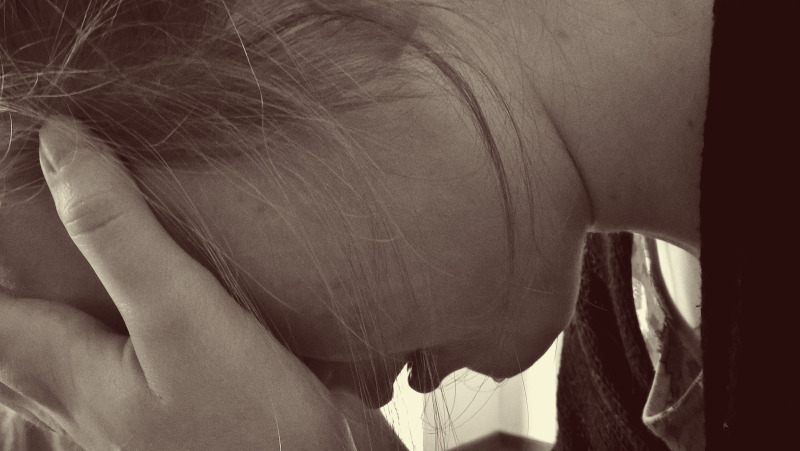
Photo: By Ulrike Mai, via https://pixabay.com/photos/woman-desperate-sad-tears-cry-1006100/.

The WHO and other UN agencies have recommended concrete actions and strategies to address GBV in the context of COVID-19 [3,4,11,12], and many countries have begun to take action to address the issue. Canada and France have provided millions in extra funding for organizations providing support to GBV victims, while Italy has released earmarked funds ahead of schedule [13]. China, Spain, and the UK have created and widely distributed guidelines for assisting GBV victims during the COVID-19 pandemic, and Chile has developed e-learning courses and counseling materials to prevent GBV [13]. Campaigns to identify and support GBV victims in pharmacies and shopping centres have been initiated in Argentina, France, and Spain, and telephone hotlines and Whatsapp services have been created or reinforced in Argentina, Portugal, Spain, and France [13].

These activities, often accompanied by media campaigns, are a step in the right direction, however much work remains to be done to protect those at risk of GBV and support victims. Specifically, further research is needed to identify the drivers of the observed increases in contact with anti-violence centers and to develop and test effective interventions to protect women at risk of GBV during the ongoing COVID-19 response.

There is also crucial need for a coordinated public health response to COVID-19 based on an intersectoral, human-rights centered framework, and science-driven theory and methods. Despite this, in Italy the current Phase 2 monitoring framework includes only 21 indicators, and none of these consider expected adverse effects of COVID-19 containment measures such as violence against women and children, mental health problems, or reduced or delayed access to health services [14]. In the absence of a comprehensive monitoring framework, whether emerging data on violence against women will actually be considered to shape future policies in Italy, or how, are still unanswered questions.

Greater cooperation is needed as well between law enforcement, health, and social services, among others, to improve surveillance of GBV and facilitate continuity and quality of care for victims [15,16]. Finally, sufficient resources must be provided for prevention and support services for GBV victims, and for data collection and research to inform effective policies.
